# *Rhipicephalus* Tick: A Contextual Review for Southeast Asia

**DOI:** 10.3390/pathogens10070821

**Published:** 2021-06-30

**Authors:** Li Peng Tan, Ruhil Hayati Hamdan, Basripuzi Nurul Hayyan Hassan, Mohd Farhan Hanif Reduan, Ibrahim Abdul-Azeez Okene, Shih Keng Loong, Jing Jing Khoo, Ahmad Syazwan Samsuddin, Seng Hua Lee

**Affiliations:** 1 Faculty of Veterinary Medicine, Universiti Malaysia Kelantan, City Campus, Pengkalan Chepa 16100, Kelantan, Malaysia; ruhil@umk.edu.my (R.H.H.); basripuzi@umk.edu.my (B.N.H.H.); farhan.h@umk.edu.my (M.F.H.R.); ibrahim.az@umk.edu.my (I.A.-A.O.); 2 Tropical Infectious Diseases Research and Education Centre (TIDREC), University of Malaya, Kuala Lumpur 50603, Selangor, Malaysia; loongsk@um.edu.my (S.K.L.); khoojj@gmail.com (J.J.K.); 3 Forest Biotechnology Laboratory, Department of Forest Science and Biodiversity, Faculty of Forestry and Environment, Universiti Putra Malaysia, Serdang 43400, Selangor, Malaysia; a.syazwansamsuddin@gmail.com; 4 Mycology and Pathology Branch, Forest Biodiversity Division, Forest Research Institute Malaysia, Kepong 52109, Selangor, Malaysia; 5 Institute of Tropical Forestry and Forest Products (INTROP), Universiti Putra Malaysia, Serdang 43400, Selangor, Malaysia; lee_seng@upm.edu.my

**Keywords:** Southeast Asia, *Rhipicephalus* tick, morphological features, tick-borne diseases

## Abstract

*Rhipicephalus* species are distributed globally with a notifiable presence in Southeast Asia (SEA) within animal and human populations. The *Rhipicephalus* species are highly adaptive and have established successful coexistence within human dwellings and are known to be active all year round, predominantly in tropical and subtropical climates existing in SEA. In this review, the morphological characteristics, epidemiology, and epizootiology of *Rhipicephalus* tick species found in SEA are reviewed. There are six commonly reported *Rhipicephalus* ticks in the SEA region. Their interactions with their host species that range from cattle, sheep, and goats, through cats and dogs, to rodents and man are discussed in this article. *Rhipicephalus*-borne pathogens, including *Anaplasma* species, *Ehrlichia* species, *Babesia* species, and *Theileria* species, have been highlighted as are relevant to the region in review. Pathogens transmitted from *Rhipicepahalus* ticks to host animals are usually presented clinically with signs of anemia, jaundice, and other signs of hemolytic changes. *Rhipicephalus* ticks infestation also account for ectoparasitic nuisance in man and animals. These issues are discussed with specific interest to the SEA countries highlighting peculiarities of the region in the epidemiology of *Rhipicephalus* species and attendant pathogens therein. This paper also discusses the current general control strategies for ticks in SEA proffering measures required for increased documentation. The potential risks associated with rampant and improper acaricide use are highlighted. Furthermore, such practices lead to acaricide resistance among *Rhipicephalus* species are highlighted.

## 1. Background

**Figure 1:**
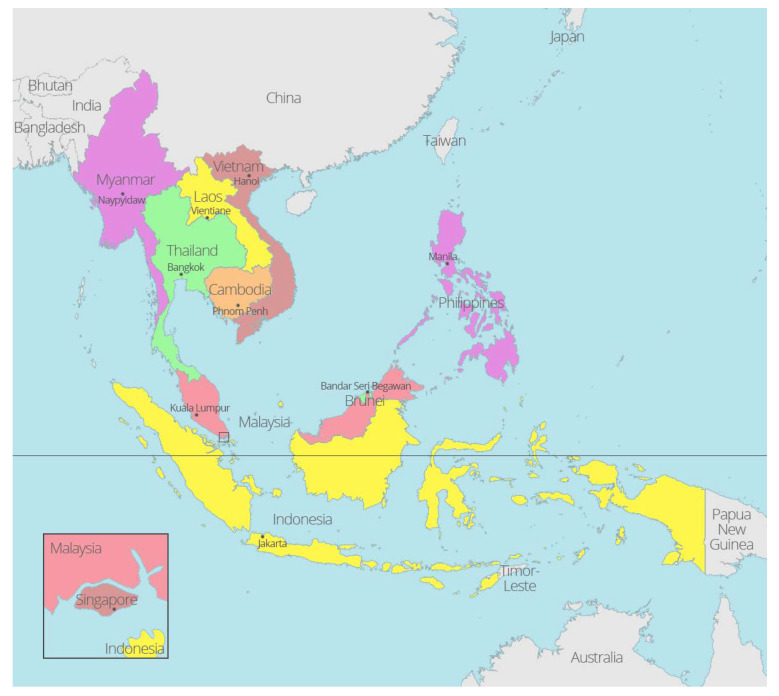
Geographic depiction of Southeast Asia: SEA comprises countries within the Indo-Chinese peninsula of continental Asia, including Myanmar (Burma), Laos, Vietnam, Thailand, Cambodia, Malaysia, Singapore, Indonesia, Timor-Leste, Brunei and the Philippines (https://aseanup.com/free-maps-asean-southeast-asia/, accessed on 4 January 2021).

Southeast Asia (SEA) covers about 4.5 million km^2^ of land mass, with a human population hovering around 670 million [1]. This region comprises 11 countries, and it is a vast Asian region situated east of the Indian subcontinent and South of China (Figure 1). All 11 countries fall within the tropical and subtropical climatic zones. The enormous variety of landscapes and climatic complexities have given rise to a considerable diversity of animals throughout the region, including ticks. With the consistent growth in the average annual gross domestic product (GDP), the concurrent expansion of SEA’s livestock sector naturally occurred [2]. Several adverse effects have accompanied this spectacular change in—the “Livestock Revolution”—the phenomenal rise in demand for foods of animal origin in society [3]. Examples include the existing threats of outbreaks of zoonotic diseases that can compromise both animal and human health [4,5], cause economic losses due to diseases [6] and result in environmental pollutions from the usage of disease control drugs and pesticides [7,8]. Small-scale livestock farming (i.e., backyard and village farms) remain the predominant practice in most low-income countries in SEA [9]. This practice requires intensive contact between livestock and farmers, which creates ideal conditions for cross-transfer of pathogens associated with potential zoonosis, in addition to ticks [10].

Ticks are second only to mosquitoes as vectors of disease of medical and veterinary importance. They transmit the widest variety of pathogens for any known arthropod vector, viz. viruses, bacteria, rickettsia, protozoa, or even certain helminths (microfilaria) [11,12]. Evidence shows that tick-related diseases are alarmingly high in the SEA region, it is understandable that the region’s climatic conditions are inherently favorable for such. Evidence shows that tick-related diseases are alarming high but have been largely ne-glected [13,14,15,16]. Although there are many academic works on ticks and tick-borne diseases (TBDs) in SEA, further to that by Petney [17], a great deal of work remains to be done [18]. Knowledge of the ticks in most countries in SEA is rather patchy or marred with ambiguity. For example, for Cambodia and Myanmar, there are no readily verifiable data on the ticks and TBDs distribution in the countries. Brunei also has meagre reports on the distribution of ticks and TBDs in companion and domestic animals. Petney et al. [18] recently compiled information on continental SEA ticks covering an extensive review of ticks and tick-borne diseases of soft ticks (Argasidae) and hard ticks (Ixodidae). In furtherance to Petney et al. [18], this review expatiates on in-depth information with a specific focus on only one genus of the Ixodidae, *Rhipicephalus*. Being the genus most frequently associated with both human and domesticated animals, *Rhipicephalus* is thus the utmost studied genus. Refined literature collections about the biology, population dynamics, importance, control and resistance issue of this hard tick in this region. This work aims to deliver a baseline for more significant works on this subject in the future.

## 2. Genus *Rhipicephalus* and Its Common Species in Southeast Asia

**Figure 2:**
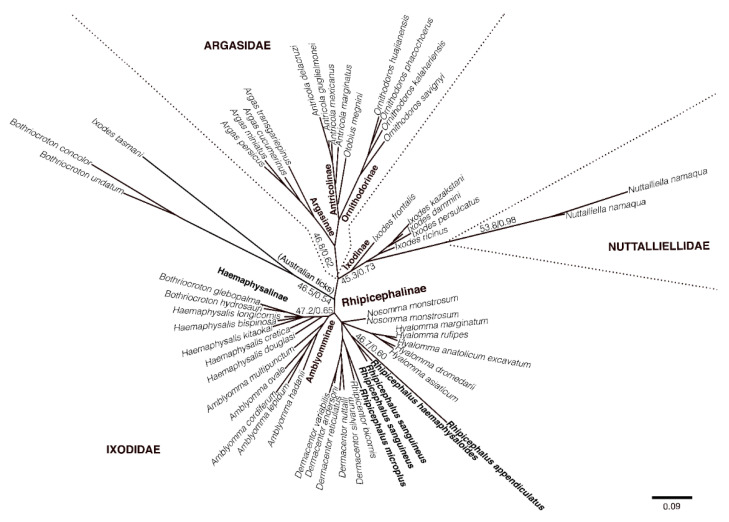
Phylogenetic tree based on maximum-likelihood analysis of the subfamilies of ticks from a 16S ribosomal RNA gene sequence alignment dataset. Branch support value on nodes indicates the bootstrap values of maximum-likelihood and Bayesian posterior probabilities. The highlighted names are *Rhipicephalus* spp. tick sequences from several countries (see Supplementary Table S1 for list of 16S ribosomal RNA gene sequence of ticks used for phylogenetic tree construction).

Ixodidae, also known as hard ticks, are exclusively parasitic arthropods. *Rhipicephalus* is one of the 12 extant genera of Ixodidae and comprises 84 described species [19,20]. *Rhipicephalus* falls under the subfamily of Rhipicephalinae (Metastriata). For this review, the phylogenetic tree of the splitting of subfamilies of ticks (suborder Ixodida) of the acarine was analyzed based on several 16S rRNA sequence representatives. The selection of genera from the subfamily Rhipiciphelinae, including the subfamilies Argasidae, Argasinae, Antricolinae, and Ornithodorinae, was analyzed with the maximum-likelihood analysis and with Bayesian analysis by using bona fide DNA sequences (Supplementary Table S1). The selection of two 16S rRNA sequences from the family Nuttalliellidae, on the other hand, is for rooting purposes as a basal lineage for this analysis. This analysis is crucial in proving the speciation of genera, which initially split among subfamilies under suborder Ixodida. This tree also shows several genera such as *Rhipicentor*, *Dermacentor*, *Hyalomma*, and *Nasomma* proximate to *Rhipicephalus* spp. and falls within the subfamily Rhipicephalinae. The phylogeny grouping of the subfamilies of ticks (suborder Ixodida) of the acarine order Parasitiformes is as follows (Figure 2).

Tick species under this genus are found globally even in regions they may not be necessarily ‘indigenous’ to. Animal trade across the SEA region and other parts of the world enhances the rapid distribution and establishment of tick species such as *Rhipicephalus*. *Rhipicephalus* species are associated with the infestation of livestock or domesticated animals, primarily cattle and dogs [18,21,22,23] imported into or exported out of the SEA region. They are mainly two- and three-host ticks (*Rhipicephalus*) or one host ticks for all the five species under *Boophilus*. *Boophilus* was so different yet similar to *Rhipicephalus* until the first strong evidence from the most comprehensive study to date. Murrell et al. [24] combined sequences from four genes (12S rDNA, COI, ITS2 and 18S) and 30 morphological characters in a “total evidence” analysis to show that the five known *Boophilus* species were a monophyletic group (99% bootstrap support) within the *Rhipicephalus* species (96% bootstrap support). The species of *Rhipicephalus* that were most closely related to the *Boophilus* species was the *R. evertsi* (Neumann, 1897) and *R. pravus* (Donitz, 1910) species-group, in which this clade consists of 13 *Rhipicephalus* species and five *Boophilus* species was well supported (99% bootstrap support). It was then proposed by Murrell and Barker [25] that the genus *Rhipicephalus* has a paraphyletic lineage with *Boophilus* and thus revised the genus *Boophilus* as synonymous to *Rhipicephalus*. However, *Boophilus* is retained as a subgenus of *Rhipicephalus*, so the synonymy of *Boophilus* with *Rhipicephalus* does not result in the loss of the name *Boophilus*, and eventually become members of *R*. (*Boophilus*).

Morphology-based taxonomic classification of *R. microplus* and *R. sanguineus* s.l. has been challenging even for the most experienced taxonomists. The intra-species variations within the *R. microplus* species complex led to the description of multiple sub-species. However, many were later considered synonyms to *R. microplus* or *R. australis* [26]. In recent years, molecular-based phylogenetic analyses added a great deal of insight into the species diversity within the *R. microplus* species complex. Based on studies of mitochondrial cytochrome *c* oxidase subunit I (COI) gene marker, there are five different phylogenetic clades within the *R. microplus* species complex viz. *R. annulatus*, *R. australis* and three *R. microplus* sensu stricto (s.s.) clades [23,26,27]. These species are not possible to be differentiated based on morphology alone. *Rhipicephalus sanguineus* s.l. on the other hand was shown to have two major phylogenetic clades, the northern (tropical) and southern (temperate) lineages [28]. Besides, several other phylogenetic clades, or operational taxonomic units (OTUs), also exist, representing separate species and needs to be confirmed in further genetic characterization [28]. Low et al. [23] revealed several COI haplotypes with high genetic differences in *R. microplus* in Malaysia. On the other hand, the genetic differences within the COI haplotypes for *R. sanguineus* s.l. in Malaysia were reported to be low [23]. Understanding the genetic diversity among these ticks is vital for vector and disease control. Specific genetic populations may show a different vectorial capacity to pathogens or even resistance to acaricides.

There are six *Rhipicephalus* species reportedly found in SEA; these include *Rhipicephalus annulatus* (Say, 1821), *Rhipicephalus australis* (Fuller, 1899), *Rhipicephalus haemaphysaloides* (Supino, 1897), *Rhipicephalus pilans* (Schulze, 1935), *Rhipicephalus microplus* (Canestrini, 1888) and *Rhipicephalus sanguineus* (Latreille, 1806) or *Rhipicephalus sanguineus* sensu lato (s.l.) [18,29,30].

*Rhipicephalus microplus* has been reported to occur in Cambodia [31], Laos [31,32], Myanmar [26], Vietnam [33,34], Thailand [35,36], Malaysia [23], the Philippines [37,38] and Indonesia [30,31]. *Rhipicephalus microplus* is frequently found on livestock animals such as cattle [30], water buffaloes [38] and goats [23]. *Rhipicephalus microplus* is widely researched as it is a significant pest of cattle with substantial economic impact [39]. *Rhipicephalus sanguineus* s.l. refers to a group of closely related species associated with dogs worldwide [40]. In SEA, it has been recorded in Laos [32,41], Myanmar [42], Vietnam [43], Thailand [44], Malaysia [45,46], the Philippines [47] and Indonesia [48]. So far, the *R. sanguineus* s.l. identified in SEA fall within the tropical lineage [45]. Nevertheless, the genetic diversity of *R. microplus* and *R. sanguineus* s.l. ticks in SEA is still largely unexplored. Not to mention that there are other species of *Rhipicephalus* whose molecular work are comparatively lesser than *R. microplus* and *R. sanguineus* s.l. *Rhipicephalus pilans*. For instance, only one nucleotide result was available in the gene bank after research on the evolution and ecological niches of *Rhipicephalus* was published in the year 2021 [49].

Despite the challenges in the taxonomy of the members of the *Rhipicephalus* genus, the existing morphological descriptions and identification keys are still crucial for assisting in the identification of at the least, species groups (i.e., *R. microplus* species complex and *R. sanguineus* species complex) for veterinary or medical diagnostic, or research purposes. Identification keys that are widely used include keys published by Walker et al. [50]. Other identification keys available for *Rhipicephalus* species found in SEA are those published by Anastos [51], Yamaguti et al. [52] and Keirans and Litvak [53]. As a common practice for identifying ticks, it may be necessary to consult more than one key for successful identification. If possible, in the identification of *Rhipicephalus* spp. the analysis of molecular barcodes should be used as complements. Genetic markers used for phylogenetic analyses, including the mitochondrial 12s and 16s rDNA and the COI gene markers, may be utilized to identify species or species complex [28,54]. Further phylogenetic analyses may be necessary for confirming the genetic lineage, depending on the research’s purpose. Some of the important morphological features used to distinguish the *R. sanguineus* species complex according to the keys published by Walker et al. [50] were based on adanal plates and conscutum of the male ticks and genital aperture of the female ticks.

In our view, the phylogenetic studies of *Rhipicephalus* lack accuracy. Therefore, instead of a few genes (in this case, 5), multiple genes should be used in combination for the analysis to be more representative and the results definitive. This combined approach would help determine whether the ticks analyzed were monophyletic or paraphyletic in a specific genetic region. This evidence is essential for establishing conclusive results on the origin and epidemiology of these voracious multiple host parasites with overlapping geographical distribution.

## 3. Host Range of *Rhipicephalus* Species in Southeast Asia

The host specificity of *Rhipicephalus* in SEA can be narrowed down based on previous incidences and findings. They are mainly associated with several types of livestock and companion animals (Table 1). Such a low variation in host species for the one-host ticks *R. microplus*, *R. australis,* and *R. annulatus* has been recorded. The three ticks parasitize only a handful of ungulate species such as cattle, goats and pigs. Three-hosts *Rhipicephalus* are the species with a broader host range, thus lower host specificity, in which they utilize different animals at different life stages. *Rhipicephalus sanguineus* sensu lato (s.l.), *R. pilans* and *R. haemaphysaloides* can be found parasitizing livestock, companion animals, wildlife and even human. Islam et al. [55] concluded that ixodid ticks are not strictly host-specific, although they might have a greater affinity for a particular host. The flexibility in ticks’ host-specificity might be one of the ticks’ survival and adaptation strategy when environmental disruptions can substantially affect the species.

The host environment’s ecological similarity may be more important than host phylogenetic similarities in increasing the survivability of the ticks [56]. For instance, intensive farming and expanding livestock production in SEA may lead to over-crowded farms and cross-infestation with ticks from one livestock species to another [18,57,58]. Nevertheless, the mark preference for one host species or high specificity of parasite-host associations is likely to result from the continual coevolution of host defenses and parasite counter-defenses that factors in the selection for reciprocal specialization [59]. For example, *R. sanguineus* s.l. is less prevalent in cats than dogs among companion animals. Other than the host preference viewpoint as reflected by this tick species’ name, cats’ intensive grooming behavior [60] might be one factor accounting for lesser tick loads in this animal [61].

Although there are six common species of *Rhipicephalus* recorded in SEA, the distribution or perhaps the information on their occurrence is not well disbursed. Indonesia is the only SEA country known to have five *Rhipicephalus* species reported with their associated hosts ranging from livestock, companion animals, rodents, and wildlife to human-documented ones. The two least reported countries are Cambodia and Myanmar. Only *R. microplus* and *R. australis* were recorded in Cambodia associated with unknown host [31,62]; while in Myanmar, only *R. microplus* and *R. sanguineus* s.l. were reportedly associated with cattle and dog. Interestingly, *R. annulatus* is seemed to be confined in Vietnam only [18]. From the literature we gathered, the most diverse species of *Rhipicephalus* can be obtained on livestock while *R. haemaphysaloides* and *R. sanguineus* s.l. seem to be exclusively associated with companion animals. Besides, *R. haemaphysaloides* is the species found infesting numerous wildlife species as compared to other *Rhipicephalus* species. To date, there are no documented tick infestations of humans and animals in Brunei [29].

**Table 1:** Host-tick list of *Rhipicephalus* hard tick in Southeast Asia.

Host Type	Country	Tick Species	Host	Reference
Livestock	Cambodia	*Rhipicephalus microplus*	Unknown	[31]
		*Rhipicephalus australis*	Unknown	[62]
	Indonesia	*Rhipicephalus australis*	Unknown	[62]
		*Rhipicephalus haemaphysaloides*	*Bos taurus* *Bubalus bubalis* *Capra aegagrus hircus*	[63]
		*Rhipicephalus microplus*	*Bos taurus* *Bubalus bubalis* *Capra aegagrus hircus* *Equus caballus* *Sus scrofa*	[30,63,64]
		*Rhipicephalus pilans*	*Bos taurus* *Bubalus bubalis* *Capra aegagrus hircus* *Equus caballus* *Ovis aries*	[30,63,64]
		*Rhipicephalus sanguineus* s.l.	*Bos taurus* *Bubalus bubalis* *Gallus gallus domesticus* *Sus scrofa domesticus*	[64]
		*Rhipicephalus haemaphysaloides*	*Bos* sp.	[32]
	Laos	*Rhipicephalus microplus*	*Bos* sp.	[32]
		*Rhipicephalus australis*	Unknown	[62]
	Malaysia	*Rhipicephalus microplus*	*Bos taurus*	[23,65]
		*Rhipicephalus microplus*	*Bos* sp.	[26]
	Myanmar	*Rhipicephalus microplus*	*Bos* sp. *Sus scrofa*	[17]
	Singapore	*Rhipicephalus microplus*	*Bos* sp. and *Bos taurus*	[36,66,67]
	Thailand	*Rhipicephalus australis*	Unknown	[62]
	The Philippines	*Rhipicephalus microplus*	*Bos* sp. and *Bos indicus* *Bubalus bubalis* *Capra aegagrus hircus*	[37,38,68]
		*Rhipicephalus haemaphysaloides*	*Bos* sp.	[69]
	Timor-Leste	*Rhipicephalus microplus*	*Bos* sp. *Capra aegagrus hircus*	[69]
		*Rhipicephalus sanguineus* s.l.	*Bos taurus*	[69]
		*Rhipicephalus annulatus*	*Bos* sp.	[70]
	Vietnam	*Rhipicephalus microplus*	*Bos* sp.	[33]
		*Rhipicephalus sanguineus* s.l.	*Bos* sp.	[71]
		*Rhipicephalus haemaphysaloides*	*Canis lupus familiaris*	[63]
Companion animals	Indonesia	*Rhipicephalus sanguineus* s.l.	*Canis lupus familiaris* *Felis catus*	[24,63,72]
		*Rhipicephalus haemaphysaloides*	*Canis lupus familiaris*	[32]
	Laos	*Rhipicephalus sanguineus* s.l.	*Canis lupus familiaris*	[41,73]
		*Rhipicephalus sanguineus* s.l.	*Canis lupus familiaris*	[45,54,74,75,76,77,78,79]
	Malaysia	*Rhipicephalus sanguineus* s.l.	*Canis lupus familiaris*	[42]
	Myanmar	*Rhipicephalus sanguineus* s.l.	*Canis lupus familiaris* *Felis catus*	[24,79,80]
	Singapore	*Rhipicephalus sanguineus* s.l.	*Canis lupus familiaris*	[28,44,79]
	Thailand	*Rhipicephalus sanguineus* s.l.	*Canis lupus familiaris* *Felis catus*	[24,38,79]
	The Philippines	*Rhipicephalus haemaphysaloides*	*Canis lupus familiaris*	[71]
	Vietnam	*Rhipicephalus sanguineus* s.l.	*Canis lupus familiaris*	[28,43,71,79]
		*Rhipicephalus haemaphysaloides*	Forest rats *	[63]
Rodents	Indonesia	*Rhipicephalus microplus*	*Rattus exulans* *Rattus hoffmanni* *Rattus rattus*	[64]
		*Rhipicephalus pilans*	*Niviventer fulvescens* *Rattus argentiventer* *Rattus exulans* *Rattus rattus* *Rattus tiomanicus*	[63,64,81]
		*Rhipicephalus* sp.	*Sundamys muelleri*	[82]
	Malaysia	*Rhipicephalus haemaphysaloides*	*Pteropus vampirus* *Rusa unicolor* *Helarctos malayanus* *Panthera tigris* *Varanus salvator* *Sus scrofa* *Hylomys suillus*	[63,83]
Wild animals	Indonesia	*Rhipicephalus microplus*	*Bos javanicus* *Manis javanica* *Rusa timorensis* *Rusa unicolor*	[63,64]
		*Rhipicephalus pilans*	*Crocidura nigripes* *Hylomys suillus* *Rusa timorensis* *Suncus murinus* *Sus scrofa*	[63,84]
		*Rhipicephalus sanguineus* s.l.	*Bos javanicus* *Rusa unicolor*	[63]
		*Rhipicephalus haemaphysaloides*	*Arctictis binturong* *Cuon alpinus* *Martes flavigula* *Neofelis nebulosi*	[85]
	Thailand	*Rhipicephalus microplus*	-	[64]
Human	Indonesia	*Rhipicephalus pilans*	-	[64,81]
		*Rhipicephalus sanguineus* s.l.	-	[63]
		*Rhipicephalus microplus*	-	[85]
	Thailand	*Rhipicephalus sanguineus* s.l.	-	[86]

* Not being explicitly mentioned on the species in the original article.

From the literature gathered for this review, it appears that while SEA countries share similar topography, the distribution of *Rhipicephalus* is not necessarily unique to any member country. The volume of works on ticks and tick-borne diseases per country is likely influenced by these parasites’ economic and health importance. Often countries with large livestock industries tend to invest more in the surveillance and research of tick-borne diseases. As earlier noted, the tropical environment of all countries in SEA renders them rich in their biodiversity. The large expanse of the region and the number of virgin forests (such as the Borneo) further emphasizes the need to explore the ecological dynamic of ticks in this region. It was proposed by Angus [87], the original invasion of Australia by *R. australis* was probably through the importation of infested cattle from Timor-Leste. A collective effort from all SEA countries in gathering this tick species’ information in SEA is essential to provide evidence on their origin, native ranges, and invasive potential. Data of *Rhipicephalus* in the countries such as Brunei and Timor-Leste that are yet to tapered are thus valuable to fill up the gaps.

## 4. The Impacts of Ticks and Tick-Borne Diseases

Animal production is considered a significant contributor to the global agricultural industry, and it plays a vital role in maintaining the food security and sovereignty of most nations. In the SEA countries, the productive and progressive livestock industry contributes to food provision and income generating for a vast population of people. The Food and Agriculture Organization (FAO) believes that the industry’s growth is driven by the growing regional economy, resilient demand, large and robust domestic market and production sectors. However, the greatest threat to the livestock industries are diseases [88]. Of relevance are diseases transmitted by ectoparasites such as ticks which adversely affects livestock productivity. In SEA, the distribution of *Rhipicephalus* across the tropical belt sustains the endemicity of tick-borne disease in the region [18].

Tick-borne diseases transmitted by *Rhipicephalus* ticks affect cattle production worldwide, including SEA countries [89,90,91]. Studies have shown the potentially devastating impact of *R. microplus* infestation on developing countries’ livestock economies [39]. These losses are bothered by developing countries’ inability to control and monitor the diseases; hence, it impairs the livestock economy [92]. The distribution and prevalence of these diseases across the SEA geopolitical area appear to be quite eco-oriented. Important *Rhipicephalus*-borne diseases in SEA are babesiosis, anaplasmosis, theileriosis, and ehrlichiosis. Some other pathogens transmitted by *R. sanguineus* s.l. include *Hepatozoon canis* [47,77,93] and *Coxiella burnetti* [76], which causes hepatozoonosis and Q-fever, respectively. The host range for these diseases is reasonably consistent, although outliers to the known host range for some tick-borne diseases have also been reported in the SEA. For instance, rare infections in a previously unknown host for *Babesia canis*, such as in wild rodents, have been reported [94] in Thailand. Similarly, Lim et al. [95] reported a rare occurrence of human babesiosis (caused by *Babesia microti*) exported from the USA into Singapore.

Babesiosis affects most warm-blooded animals with high economic and health consequences. In SEA, *Babesia vogelli*, *B. gibsoni* and *B. canis* transmitted by *R. sanguineus* s.l. appears to be the most prevalent in dogs [47]. In contrast, *B. bigemina* and *B. bovis* are prevalent in ruminants [96,97,98]. Of importance are *B. microti* (USA and Kobe types) and *Babesia BiCM002* in rodents [99]. Whether *Rhipicephalus* transmits these *Babesia* species in rodents is yet to be understood. Thus, the possibility of a rodent-to-human cycle for *B. microti* in SEA needs to be investigated. Such an investigation would help determine the impact of *B. microti* infection in the epidemiology of babesiosis in SEA countries. The prevalence of babesiosis is variable amongst species and breeds but tend to be lower in well-managed farm settings or companion animal care. The prevalence of bovine babesiosis appears to have dropped in Indonesia from 96% in 1993 [100] to 69.8% in 2017 [98]. The reason for the drop has been attributable to improved veterinary and herd health management. Similar approaches are in place in Malaysia under the Department of Veterinary Services’ purview, requiring a rigorous overview of the recent program outcome.

*Babesia caballi* and *Theileria equi* collectively cause equine piroplasmosis characterized by fever and jaundice, mainly in horses and other Equidae in SEA [101,102]. Horses infected with *T. equi* are known to remain seropositive for the rest of their lives [103]; hence should be promptly filtered out during surveillance. *Theileria orientalis* (also called *T. buffeli* and *T. sergenti*) appears to be the most prevalent *Theileria* species of ruminants transmitted by *Rhipicephalus* in Cambodia, Vietnam, and the majority of SEA. Malaysia witnessed a 68–72% prevalence among the young and adult cattle population in Selangor [104]. The trend seems to have risen exponentially from 0.1% to 2.0% prevalence for Malaysia in 1990 [105]. *Theileria sinensis* and *Anaplasma platys* were recently detected in a Malaysian cattle population presented clinically with normocytic normochromic anaemia [106].

*Anaplasma* that causes anaplasmosis is a tropical to subtropical rickettsial disease of ruminants and companion animals. *Anaplasma marginale* and *A. centrale* are the notable species in cattle and buffaloes across SEA [107], while *A. platys* occur in dogs [93,108]. Diagnosis of rickettsial diseases in this region has been challenging over the years, which call for a didactic approach and increased awareness. The epidemiological dynamics of rickettsial infections in humans and animals remain under-investigated in SEA [109], thereby exposing the SEA terrain to sustained infection without a viable control or surveillance policy. The role of *Rhipicephalus* in the propagation of these diseases need to be established.

Ehrlichiosis is of high economic and health importance in dogs and immunocompromised handlers or owners. *Ehrlichia canis* is most prevalent in dogs, causing a febrile disease characterized by a severe multi-systemic inflammatory response. *Rhipicephalus sanguineus* s.l. is the primary vector for transmission of ehrlichiosis. *Ehrlichia canis* affects monocytes and causes canine monocytotropic ehrlichiosis, while *E. ewingii* infects granulocytes to cause canine granulocytic ehrlichiosis [110]. Thus, this genus is also of medical importance (affecting human beings). A recent study reported high antibodies titre for *E. chaffeensis* antigens among the indigenous people in Malaysia (34.3%) and animal farmworkers (29.9%). However, no *E. chaffeensis* DNA was detected from any tick vectors; instead, *Ehrlichia sp*. strain EBm52, *E. mineirensis* and “*Candidatus E. shimanensis*” were detected in *R. microplus*, and *H. bispinosa* collected from cattle [111].

Currently, tick-borne protozoal and rickettsial diseases are invariably endemic in SEA. Concurrent infectious diseases with *Babesia*, *Theileria*, *Anaplasma* and *Ehrlichia* spp. are increasingly reported. The theory of increasing sensitivity of pathogens detection with the help of molecular work could logically fit this scenario. However, it remains unclear why such co-morbidities are consistently challenging to treat, and the ticks are difficult to control in the environment. Hence, an elaborate effort is required to identify the epidemiological patterns of *Rhipicephalus*, the pathogens they transmitted and the rising incidence of resistance to control drugs of this tick in SEA. Molecular detection of the presence of pathogens in squashed ticks is more direct in understanding the host-parasite dynamics for TBDs should be extended further to involve more host species of *Rhipicephalus* in the region. It remains crucial to determine the extent to which *Rhipicephalus* species act as biological, mechanical vectors or both for pathogens of interest.

Tick-borne protozoan diseases cause substantial economic loss in Thailand’s dairy and beef industries [112]. High mortality rates were noticed in the 50 million USD imported exotic breed of cattle due to tick-borne diseases. The Department of also expended over 20 million USD to diagnose, treat and control diseases of animals. However, the exact economic impacts of ticks and tick-borne diseases in SEA are not available due to the lack of farm economic impact study compared to the European and African regions [113]. The most recent studies available on the impacts of ticks with estimated figures were from the major meat producer countries like Brazil, Tanzania and Mexico, which suffered losses in meat production up to 3.24 billion USD per year [39]; 364 million USD per year [114] and 573 million USD per year [115] respectively. Limited data on the economic losses due to ticks and tick-borne diseases are clearly stated in a paper from the Philippines [116]. The absence of a formal financial losses report could have led to the ignoration by the government on the implementation of tick control in the routine animal health program in most SEA countries.

Information on the economic impact of tick and tick-borne diseases, including losses and control of tick-borne diseases in Indonesia (3.1 million USD annually) and the Philippines (0.6 million USD annually), dates back to 1999. These data were calculated by McLeod and Kristjanson [117] by using a spreadsheet model (Tick Cost). An initiative was taken to estimate the losses attributable to ticks in SEA countries (Supplementary Table S2) to highlight ticks and tick-borne diseases’ economic importance. This estimation was based on the studies on the losses from the average milk production (90.24 L per cow) [118] as well as the average financial loss (production losses plus the cost of control) per animal per year (7.3 USD/head/year) [119]. Overall, the estimated financial loss and milk loss in the cattle industry due to ticks and tick-borne diseases is 63 million USD and 470 million liters or equivalent to 159 million USD, in the year 2019. With limited information on the percentage of cattle at risk of tick infestation, the breed of the cattle, and the tick burdens for each cattle breed from this region, this estimation is relatively crude. Besides, the economic impact of direct losses due to tick-borne diseases related to mortality or abortions is inaccessible due to data scarcity in the region.

In estimating *Rhipicephalus*-related economic losses in the animal industrial sector, attention should be directed towards the different levels of management. The levels of tick management include tick control by fumigation or use of acaricides in the form of dips, injectables or pour-on. The cost of tick control and resistance, in addition to that, should be enumerated. Similarly, resistance to anti-protozoal and anti-rickettsial are significant factors to be considered in the quantification of losses. Antiprotozoals, including diminazene aceturate and imidocarb, are frequently used in the SEA region. Hence there is a resultant increase in the incidences of resistance [120]. Other considerable economic variables are the thriftiness of animals affected by *Rhipicephalus* infestation or *Rhipicephalus*-borne diseases.

## 5. Resistant and Susceptibility Host Responses

The complex interaction, mainly due to the host’s diverse immune mechanisms and non-immune structural components, has contributed to various responses towards tick feeding [121]. Most mammals mount an immunological response to a feeding tick bite. It is often more vital to the host’s species with little or no evolutionary experience. Some species or breed appear to be better adapted to the tick bite; for instance, *Bos indicus* cattle breeds are more resistant to *R. microplus* than *B. taurus* breeds, although considerable variation in resistance exists between and within breeds [122]. The pattern of host resistance to ticks in the SEA region is not necessarily different from other parts of the world. Such resistance is often dependent on the commonality of the several species. Resistance is generally believed to be under genetic control [123]; thus, highly resistant animals can be selected to progress genetic improvement in tick resistance within a herd. In 1997, tick resistance and the effects of dexamethasone and anti-histamine were investigated in four Kedah-Kelantan (KKKK), four FI Kedah-Kelantan X Friesian (KKFF), and four 25% Kedah-Kelantan X 75% Friesian (KFFF) using experimental tick infestations in Malaysia [124]. While in Thailand, a study has been done by Kongsuwan [123] to gain insights into the molecular basis of host resistance that occurs during *R. microplus* attachment. These are the two available studies on resistant and susceptibility host responses done on the domesticated cattle in SEA [123,124]. The study on tick-host interactions remains scanty in SEA. Therefore, works in this section are a general context with aspiration; it could provide insight for further research in this area for the common breeds in this region.

*Rhipicephalus microplus* is the most studied ticks in host responses, with several factors that have been identified as influential to the resistance of cattle to *R. microplus* [10,121,125,126,127]. Such factors include grooming behavior [128]; innate immunity response which involve histamine secretion [129], mast cells and basophil hypersensitivity reaction at the tick bite sites [130,131,132] and intra-epidermal vesicles that contain mainly neutrophils to prevent attachment of larvae or forcing them to detach [121]; adaptive immunity which implicate IgG1 antibodies and sera of the host [133]; and lastly physical defenses whereby the skin features, vascular architecture and hemodynamics such as the dilation of arteriovenous and anastomoses in the skin playing a vital role in tick rejection [121,123,131]. All of the above mechanisms will lead to failure of tick attachment and low feeding rate, therefore increasing the chances of tick removal by grooming behavior of the animals when the ticks need to spend more time trying and looking for feeding sites.

Overall, resistance to *R. microplus* infestation in cattle has many effector mechanisms. Although some of the mechanisms and modulating factors have been identified and quantified, much remains to be explained. Studying the genetic resistance to ticks among different breeds of cattle can contribute to alternative control methods. Investigations have intensified the crossing of these two groups, aiming to obtain more resistant animals to the conditions found in tropical countries and are also good meat producers. Regarding SEA, in addition, the host-range resistant factors should be expanded to include companion animals, wild animals, and livestock to understand the phenomenon. For future research, potential research of wild cattle in SEA such as Banteng (*Bos javanicus*), Gaur (*Bos gaurus*) and water buffalo (*Bubalus bubalis*) can be explored for conservation and genetic diversification purposes.

## 6. Controlling and Acaricides Resistance

According to the records in “Arthropod Pesticide Resistance Database—https://www.pesticideresistance.org, accessed on 4 January 2021”, *R. microplus* has been considered the most pesticide-resistant tick to date. *Rhipicephalus microplus* resistance to at least 50 active ingredients have been documented worldwide; *R. sanguineus* s.l., on the other hand, is being recorded to be resistant towards five active ingredients. Unfortunately, research on acaricides efficacy against *Rhipicephalus* ticks in SEA is not available to the best of our knowledge. Evidence in the current literature on the methods of tick control in SEA is also somewhat limited. This review raises awareness for the potential development of tick control initiatives in countries of this region.

*Rhipicephalus* ticks’ control mainly depends on conventional acaricides. However, the exhaustive use of these chemicals has resulted in tick populations developing resistance to major acaricide chemical classes [134]. Ivermectin, a macrocyclic lactone, is used as an endo-ectoparasiticide. It is used as an acaricide and anthelmintic in goat and sheep farms in Malaysia [135], Indonesia [136], and Thailand [137]. Although there is currently no report of acaricide-resistant *Rhipicephalus* ticks in the SEA region, we cannot discount the possibility of this event. Thus, the application of alternative tick control approaches, including the rotation of acaricide, sterile hybrid ticks, pasture rotation, anti-tick vaccine, development of host resistance to ticks and the use of plant extracts, should be explored in SEA.

The alternation of the use of two or more acaricide with different modes of action could be an advantageous tick control method as well as a measure to prevent cross-resistance [134]. Amitraz is an example of an acaricide that could effectively be employed in an acaricide rotation strategy. There is evidence of the loss of resistance to amitraz in populations of ticks on farms where the cattle were treated with other alternative acaricides [134]. The loss of resistance to amitraz after treatment of *R. microplus* infested calves with spinosad in rotation with amitraz resulted in the loss of amitraz resistance in Australia [138]. However, these promising laboratory findings must be field-tested before any acaricide rotation strategy can be implemented in SEA.

The success of mosquito control using genetic control methods [139] rekindled interest in using this method to control *Rhipicephalus* ticks. Osburn and Knipling [140] demonstrated sterile males’ production and fertile females through the mating between *R. annulatus* and *R. microplus*. The backcrossing of fertile female progenies also produces sterile males and fertile females [140]. However, several considerations have to be studied before this method is implemented. Firstly, it is costly to produce sterile hybrid males. Secondly, the sterile hybrid males can only be dispersed over a small area to ensure that they find the opposite sex easily. Lastly, the sterile hybrid males must be affirmed as harmless to humans before field release [141].

The per capita consumption of livestock products among SEA countries is projected to increase in the years to come [142] significantly. The increase in demand for livestock products has intensified the race to acquire agricultural land between the livestock and crop farmers. Integrating both cash crop plantations with ruminant cultivation is very much encouraged [143]. In Malaysia, cattle can graze in oil palm plantations to reduce wild weeds [143]. In Laos, smallholders also practice similar methods to manage crop and cattle production [144]. Using the pasture rotation method, the principle is to starve larval ticks by rotating cattle into ‘clean’ areas at specified intervals [134]. However, this method had limited appeal to cattle producers because of the laborious management and the possible adverse effect on pasture quality [143]. A recent study in Brazil found that pasture rotation may not sufficiently decrease the burden of *R. microplus* on host cattle [145], making this method unappealing.

Since the excessive use of acaricides has been shown to cause the accumulation of chemical residues in milk, meat, and the environment, safer methods have arisen. Vaccination or immunological control is touted as the most promising, environmentally friendly, and sustainable strategy for the management of *Rhipicephalus* infestation [146]. Bm86-based vaccines have been successfully applied under field conditions and can induce cross-protection against several tick species [146,147]. However, the presence of *R. microplus* showing low susceptibility to Bm86-based vaccine prompted researchers to examine other additional antigens such as the Bm95 that could evoke protection against broader tick species [148]. Besides, the concept of cocktails vaccines has been mooted to enhance effectiveness and impact more comprehensive tick species [146]. The idea of having cocktail vaccines with different antigens working in synergistic tandem to attack other physiological processes of various tick species is undoubtedly something worth anticipating. These vaccines will benefit countries in SEA where sometimes, different animals (livestock, wildlife, pets and companion animals) may live close to one another and may be infested with ticks of various species [142,149].

Host resistance to ticks is an essential factor affecting tick control economics as it is a low-cost, permanent solution that requires no extra resources [150]. The host resistance phenotype is also heritable to a certain extent [151]. However, the main reason for the lack of development of this solution is the difficulty in identifying individual-animal variation in resistance to ticks and the cost involved [152]. In general, cattle farming in SEA is populated by *Bos indicus* breeds [153,154]. These breeds have been shown to have a higher resistance to tick infestations than other cattle breeds [152]. Additionally, resistance to either one of the tropical stressors, such as the resistance to ticks, worms, or heat stress, positively correlates to the other stressors, suggesting that the genes’ expression is interrelated [155]. It would be advantageous for genomic selection of desirable traits to be performed on *Bos indicus* breeds that are resilient to the tropical climate of SEA.

Plant extracts or secondary metabolites, including flavonoids, terpenes, spilanthol and coumarins, have been studied comprehensively for their potential to control ticks [156]. The primary reason for the development of plant extracts for tick control is the global inclination to reduce chemical acaricides for fear of developing resistance and the presence of chemical residues that pose a danger to human and animal health [156]. SEA is home to the diverse biodiversity of tropical plants that have been shown to possess medicinal properties [157]. *Petiveria alliacea* is commonly found in SEA [157]. Rosado-Aguilar et al. [158] showed that crude extracts and fractions from stems and leaves of *P. alliacea* have acaricidal activity against larvae and engorged females of *R. microplus*.

Additionally, *R. microplus* larvae were decimated entirely by using *P. alliacea* methanolic extracts [158]. Besides, turmeric oil (curcumin) was used to prevent tick bites for dogs [159]. Finally, extracts from *Citrus hystrix* and *Cymbopogon citratus* have acaricidal activity against *R. microplus* larvae [160]; these plants are commonly used in food preparation SEA.

In essence, livestock farmers in SEA are the most burdened by problems associated with *R. microplus* infestation. However, due to the structural issues plaguing the SEA livestock industry (such as the high cost of animal feeds, lack of quality breeds, inefficient coordination of agricultural policies and limited industry linkages [161,162,163,164], most smallholder farmers resort to using acaricide as it is the most cost-effective method to control tick infestation. Hence, in addition to structural reforms to the agriculture policies by the respective governments, farmers must be educated on sustainable agricultural practices and shown the impact of such practices in improving income levels [165]. Besides, there should be more university-industry-farm partnerships for the pilot-testing of newer technologies such as the application of Internet-of-Things and artificial intelligence to improve aspects of livestock farming [161]. This concept of smart farming was tested by the collaboration between True Corporation, Charoen Pokphand Foods and Khon Kaen University, Thailand, on the application of 5G technology and AI in monitoring livestock health [161]. Other *Rhipicephalus* tick control efforts in SEA include developing the promising Bm91 vaccine based on Thai *R. microplus* strains. These strains showed a long-lasting immune response in immunized cattle [166] and the simple method of immersing tick-infested cattle in water for as short as 5 min to alleviate tick burden in Thai cattle [167].

## 7. Conclusions

The *Rhipicephalus* species is abundant and widely distributed in SEA. There seems to be no propensity for certain *Rhipicephalus* species in one SEA country over another because of the uniformity in environmental parameters. Thus far, the host range for *Rhipicephalus* is within those animal species of domestic reach (from food animals to companion animals to rodents). The presence and host range of *Rhipicephalus* species in the wild is yet to be studied and understood. There is a realm of unknown ecodynamics for this species. Nevertheless, *Rhipicephalus pilans* were found in some wild animals in Borneo. The distribution in other countries and domestic animals need crucial investigation to factor in this species in the epidemiology of tick-borne diseases in the region. The occurrence of ticks and tick-borne diseases in SEA follows a trend of the countries’ affinity for specific domestic species and outbreak incidence. Those with a higher buffalo population, such as Thailand and Cambodia, would have a higher report of *Rhipicephalus* and TBDs prevalence associated with buffaloes, and vice versa for countries that farm cattle or small ruminants more.

Tick-borne diseases in SEA remain poorly characterized, mainly due to limited expertise and insufficient research interest. Base on the works collected from this review paper, we found that the knowledge of *Rhipicephalus* ticks in this region is still somewhat restricted. Reports and studies of these ticks focused primarily on the occurrence and the diseases associated with this parasite. Even though this genus of ticks consists of the two most economically important species, the data on their impacts in both the livestock and pet industry in SEA countries are not available. In some countries, there are absolutely no reports. Therefore, concerted efforts must be mounted to establish a rapporteur system for tick and TBDs in SEA. Babesiosis, anaplasmosis, and theileriosis are the most reported tick-borne disease of animals in SEA. Diagnosis is usually based on clinical signs of anemia, jaundice, fever, and laboratory findings, while treatments range from antibiotics to antiprotozoals. The roles the *Rhipicephalus* plays in the potential mechanical transmission of these diseases remains unclear even as the biological vector status is established.

The poor availability of epidemiological (and ecological) observations on *Rhipicephalus* ticks in these countries is the key constraint of developing our understanding of the economic impacts either due to direct loss (mortality/morbidity) or indirectly due to the costs for controlling this parasite. Acaricides resistance is also one of the fields that required much work to be done for these regions. Acaricide resistance was being reported back to 1967 in Malaysia, and this was the only report available to date to the best of our knowledge. Despite clients’ complaints about acaricides resistance being heard sporadically, there is no research investigating the depth of the effect. There is also limited work on the scientific work being conducted to assess the efficacy of these available acaricides towards the *Rhipicephalus* ticks in this region. Despite the lack of all the above mentioned, efforts on seeking alternative controls for these ticks are being carried out, with the majority focus on plant extracts. As mentioned earlier, works done on *Rhipicephalus* ticks are selective; most surveillance systems are only conducted on livestock surveillance and are not well linked to human or wildlife surveillance. There is, therefore, a gap from the aspects of the human-livestock-wildlife interface that need to be filled. The scantiness of information for *Rhipicephalus* ticks in SEA is undoubtedly an issue that needs to be tackled. With 171 million pigs and ruminants to feed over 620 million inhabitants and USD 1412.7 million worth of pet market in SEA, the unspotted threats from the ticks might be more significant than what we expect.

Undoubtedly there is a wide gap of information about *Rhipicephalus* beyond those known for the domestic animals. An extensive survey for the urban and sylvatic relationship between tick, host and pathogens is needed to cover the information gap. At the same time, the phylogenetic diversity of the known ticks should be studied further in SEA.

## Data Availability

Not applicable.

## Supplementary Materials

The following are available online at https://www.mdpi.com/article/10.3390/pathogens10070821/s1, Table S1: List of 16S ribosomal RNA gene sequence of ticks used for phylogenetic tree construction in Figure 2, Table S2: Economic losses.
