# Supplementary material for: Rhipicephalus Tick: A Contextual Review for Southeast Asia
Source: Pathogens. 2021 Jun 30;10(7):821. doi: 10.3390/pathogens10070821 (PMC8308476; doi:10.3390/pathogens10070821)
Supplement: Supplementary file 1 [file pathogens-10-00821-s001.zip › pathogens-1201099-supplementary.pdf]

Supplementary files (Table S1 & S2)

Table S1. List of 16S ribosomal RNA gene sequence of ticks used for phylogenetic tree construction in Figure 2

Family	Subfamily	Species	Country	Isolate/ strain/ voucher	Accession No.
Argasidae	Argasinae	*Argas cucumerinus*	Chile	Arcu	KX258879
		Argas miniatus	Brazil	DF2	KY705381
		Argas persicus	China	GS	MK555333
		Argas transgariepinus	South Africa	Koegas	MG941007
	Ornithodorinae	*Ornithodoros huajianensis*	China	AMMSC-T-10830	MK208994
		Ornithodoros kalahariensis	South Africa	Obra 2_1	MF415636
		Ornithodoros phacochoerus	South Africa	Oknp 2_1	MF415621
		Ornithodoros savignyi	South Africa	Osud 2GB	MF415646
	Antricolinae	*Antricola delacruzi*	Brazil	n/a	EU090906
		Antricola guglielmonei	Brazil	n/a	EU090905
		Antricola marginatus	USA	n/a	L34324
		Antricola mexicanus	USA	n/a	L34323
		Otobius megnini	Argentina	n/a	EF120989
Ixodidae	Amblyomminae	*Amblyomma cordiferum*	Malaysia	T26	MK301096
		Amblyomma hadanii	Argentina	n/a	KJ584370
		Amblyomma ovale	Argentina	n/a	AF541255
		Amblyomma lepidum	Israel	n/a	KP987777
		Amblyomma multipunctum	Ecuador	n/a	KC677673
	Haemaphysalinae	*Bothriocroton concolor*	Australia	n/a	JN863727
		Bothriocroton glebopalma	USA	n/a	U95859
		Bothriocroton hydrosauri	USA	n/a	U95861
		Bothriocroton undatum	Australia	n/a	AH011488
		Haemaphysalis bispinosa	India	Chipahikhola	MF946461

	Haemaphysalis cretica	USA	n/a	L34308
	Haemaphysalis douglasi	Japan	HD-B	AB819176
	Haemaphysalis kitaokai	China	Haemaphysalis_T5124-2	MH208573
	Haemaphysalis longicornis	China	SZ6	KJ710106
	Hyalomma anatolicum excavatum	India	Z9	KP210048
Rhipicephalinae	*Dermacentor andersoni*	Canada	n/a	FM955615
	Dermacentor nuttalli	China	Neimeng	MK032591
	Dermacentor reticulatus	Spain	1GV09/25	JF791811
	Dermacentor silvarum	China	NX-guyuan	MK032807
	Dermacentor variabilis	Canada	n/a	FN665384
	Hyalomma asiaticum	China	IM048	JX051087
	Hyalomma dromedarii	USA	n/a	L34306
	Hyalomma marginatum	USA	n/a	L34307
	Hyalomma rufipes	South Africa	Hrufi9	KU130464
	Nosomma monstrosum	South Africa	Nmons1	KU130405
	Nosomma monstrosum	South Africa	Nmons2	KU130406
	Rhipicentor bicornis	USA	n/a	L34304
	Rhipicephalus appendiculatus	Uganda	UG-3	KY688462
	Rhipicephalus sanguineus	Taiwan	16S+1.L	DQ093297
	Rhipicephalus sanguineus	USA	4981	MH018842
	Rhipicephalus microplus	India	T44	KP210071
	Rhipicephalus haemaphysaloides	Thailand	n/a	KC170743
	Ixodes tasmani	Australia	B6	MH043269
	Ixodes frontalis	Brazil	Z1	KP769862
	Ixodes ricinus	Canada	CA4	HG916809
	Ixodes kazakstani	Kyrgyzstan	481-1987	MF095803

		Ixodes persulcatus	Sweden	3TJ	KX384801
		Ixodes dammini	China	IPS-F8-F	DQ093299
Nuttalliellidae	Nuttalliella	*Nuttalliella namaqua*	South Africa	Nn_Gut_Liz4	JF729313
		Nuttalliella namaqua	South Africa	Nn_Gut_Liz8	JQ739172

Table S2. Economic losses

Country	Cattle (head) [a]	Milk Cow (head) [b]	Financial Loss (USD) [a x 7.3 USD]	Milk Loss (litres) [c] [b x 90.24 litres]	Potential Milk Loss (USD) [c x 0.34 USD*]
Brunei	4,752	106	34,690	9,565	3,252
Cambodia	469,542	121,627	3,427,657	10,975,620	3,731,710
Indonesia	2,498,083	561,061	18,236,006	50,630,145	17,214,249
Laos	280,937	44,879	2,050,840	4,049,881	1,376,959
Malaysia	105,923	41,599	773,238	3,753,894	1,276,323
Myanmar	1,811,211	3,900,000	13,221,840	351,936,000	119,658,240
Singapore	77	-	562	-	-
Thailand	907,093	220,000	6,621,779	19,852,800	6,749,952
The Philippines	881,212	5,420	6,432,848	489,101	166,294
Timor-Leste	11,124	-	81,205	-	-
Vietnam	1,794,421	317,729	13,099,273	28,671,865	9,748,434
Total	8,764,375	52,124,21	63,979,938	470,368,871	159,925,416

Source: FAOSTAT 2019 (http://www.fao.org/faostat/en/#data/QL)

* Milk priced average 0.34 USD/litre in 2019 according to trading on a contract for difference (CFD) that tracks the benchmark market for this commodity (5 March 2021).
